# Application of 3D Bioprinting in Urology

**DOI:** 10.3390/mi13071073

**Published:** 2022-07-07

**Authors:** Yue Zhao, Yuebai Liu, Yi Dai, Luo Yang, Guo Chen

**Affiliations:** 1 Department of Urology, West China School of Public Health and West China Fourth Hospital, Sichuan University, Chengdu 610000, China; zy_0818945@163.com (Y.Z.); zy_no2_wjkhh@163.com (Y.D.); 2 Department of Public Health Laboratory Sciences, West China School of Public Health and West China Fourth Hospital, Sichuan University, Chengdu 610000, China; 3 Department of Education and Training, Sichuan Cancer Hospital, Chengdu 610000, China; liuyuebai0611@163.com; 4 Laboratory of Reconstructive Urology, Institute of Urology, West China Hospital, Sichuan University, Chengdu 610000, China

**Keywords:** 3D bioprinting, tissue engineering, urology

## Abstract

Tissue engineering is an emerging field to create functional tissue components and whole organs. The structural and functional defects caused by congenital malformation, trauma, inflammation or tumor are still the major clinical challenges facing modern urology, and the current treatment has not achieved the expected results. Recently, 3D bioprinting has gained attention for its ability to create highly specialized tissue models using biological materials, bridging the gap between artificially engineered and natural tissue structures. This paper reviews the research progress, application prospects and current challenges of 3D bioprinting in urology tissue engineering.

## 1. Introduction

Damages to urinary tissues and organs, such as trauma, tumors, congenital malformations, birth injuries and ageing, remain a major clinical challenge. The worldwide shortage of organs and the scarcity of donors has sounded the alarm for alternatives to the allogeneic transplantation of tissue. However, in traditional tissue engineering, there are still significant problems, such as the inhomogeneous distribution of cells, the loss of structural integrity and difficulties in vascularization of lager and thicker tissues [1,2]. As a potential solution, 3D bioprinting may overcome some of the aforementioned challenges associated with tissue engineering. On the one hand, 3D bioprinting will enable a better understanding of the pathology mechanism and favor the discovery of novel therapies for early intervention. On the other hand, 3D bioprinting can stimulate the body′s natural organs and tissues to replace those end-stage or severe urology diseases [3]. 

**Figure 1:**
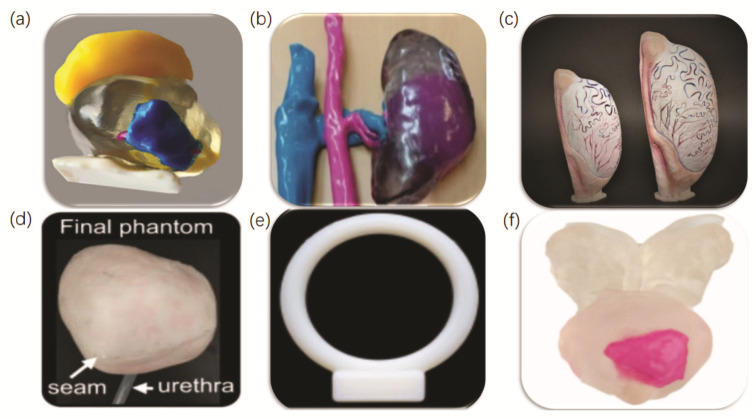
Three-dimensional-printed urological models for training and education: (**a**) 3D-printed prostate cancer model [7]; (**b**) 3D-printed kidney cancer model [8]; (**c**) 3D-printed testis model [9]; (**d**) 3D-printed bladder model [10]; (**e**) 3D-printed thermoplastic elastomer (TPC) urethra pessary model [11]; (**f**) 3D-printed prostate model [12].

Three-dimensional printing, first described in the 1990s, has found an increasing application in all fields of urology. Initially, 3D printing technology begun to influence the field of urologic surgery, from creating 3D models for medical stuff training, surgical planning and patient education to manufacturing implants and personalized prostheses [4,5,6]. The 3D-printed urological models are presented in Figure 1.

**Figure 2:**
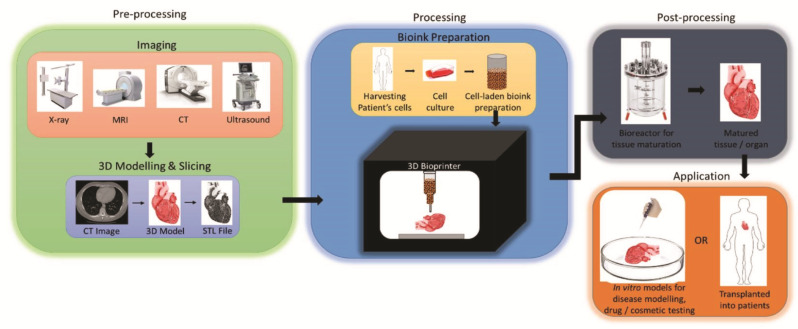
Schematic illustration for the process of 3D bioprinting. Reprinted with permission from ref. [20].

The shortage of organs and accurate tissue models for medical use resulted in the birth of 3D bioprinting. Broadly speaking, 3D printing related to the direct biomedical field can be regarded as 3D bioprinting. Most estimates point to a global 3D bioprinting market worth more than USD 1 billion in the near future [13,14]. As Figure 2 shows, 3D bioprinting is based on computer 3D models combined with several kinds of cells, scaffolds and biomolecules, and prints the required biomedical products according to the dispense ‘bioinks’ function and environment [15,16]. Currently, according to different prototyping principles and printing materials, the 3D bioprinting technology is mainly divided into three types, including inkjet 3D bioprinting, micro-extrusion 3D bioprinting and laser-assisted 3D bioprinting. Each technique has its technical characteristics, as summarized in Table 1 [15,16,17,18,19].

## 2. The Methods of 3D Bioprinting

### 2.1. Micro-Extrusion 3D Bioprinting

Micro-extrusion bioprinting is the most widely used bioprinting method based on the principle of physical extrusion of bioink droplets for the desired 3D structures [15,16]. The biomaterials used for this type of printing are usually fixed in micro-scale nozzle holes or micro-pin holes on the substrate. The fluid and dispersion of continuous filaments are then formed by coordinated extrusion movements caused by pneumatic pressure, plunger pressure or screw pressure. In addition, this method can print a wide range of “bioinks” with high biocompatibility and have the potential to print anatomical porous structures. However, this technology also has some disadvantages, such as limited printing accuracy and lower activity during the printing process [21,22].

### 2.2. Inkjet 3D Bioprinting 

Inkjet 3D bioprinting functions as a non-contact printing process, where biological ink droplets fall at specific locations on the surface of the print carrier as required by the 3D model. The advantages of inkjet printing include low overall costs, fast printing speeds and high resolution [14]. Nonetheless, there are several limitations, including the uneven droplet size and disorder and the frequent nozzle clogging [18,19].

### 2.3. Laser-Assisted Bioprinting

Laser-assisted bioprinting (LAB) uses monochromatic pulses or continuous laser energy to transfer biological materials in the form of droplets from a donor slide to a collector slide, resulting in the creation of 3D structures [13]. To date, the ultrafast laser induction of animal cell hydrogel has been developed by the femtosecond laser. LAB prints all cell types without nozzles and causes no cell blockage and the bioinks with cell densities that are close to physiological tissues or organs; thus, the cell survival rate and the resolution ratio of this method are considerable. Nevertheless, the clinical application is limited by the easily contaminated absorption layer and high costs [15,16,23,24]. 

## 3. The Bioinks of 3D Bioprinting

The materials utilized for 3D bioprinting are called “bioink”. The diverse properties of different tissues or organs have a specific ECM with a varying composition that supports cellular growth and function. The ECM is the non-cellular scaffolding framework for tissues and organs, representing an environmental niche in which cells live, proliferate, migrate or modify their phenotype [25,26,27]. Therefore, researchers must reproduce the diverse ECM of natural tissues and organs in the process of 3D bioprinting and the commonly used printing inks are decellularized ECM, hydrogel and 3D porous bio-scaffold. The composition, classification and characteristics of each bioink are illustrated in Table 2.

### 3.1. Decellularized ECM

Naturally sourced ECM is the most suitable printing material for the growth and differentiation of human cells because of its similar environment to the normal tissue structure and microenvironment. The decellularized extracellular matrix (dECM) removes the cellular components and some small molecules or antigens; thus, it has better biocompatibility, degradability and immunogenicity than naturally sourced ECM. Moreover, the dECM retains most of the native structure and composition of the tissue or organ, such as growth factors, polysaccharides and natural proteins that are critical to promote angiogenesis and the growth and differentiation of cells, providing strong mechanical supports and a suitable living environment for cells [28].

In the existing research, one major source of dECM bioinks comes from pigs [29,30,31]. Rat and goat tissues and organs, such as adipose tissue, heart and liver, are additional sources of dECM bioinks [32,33,34]. Nevertheless, it is important to point out that several viral genomes are integrated into animal genomes, making the use of animal tissues and organs a risk [35], which requires our attention. Additionally, several groups use cadavers and donated human parts as a source of dECM for graft development [36,37,38]. Human adipose tissue is also considered a good choice of dECM bioinks and stem cells, and patients who normally undergo liposuction can produce a lot of medical waste in the form of adipose tissue that can be turned into useful biomaterials [39]. Recent studies have also shown that plant-derived tissues are suitable candidates for vascularization in tissue engineering for their interactive network structure, large surface area, varying degrees of hydrophilicity and superior mechanical properties [40].

The process of decellularization can be prepared by several chemical, biological and physical methods [41,42]. Ammonium hydroxide, detergents, SDS, acids and bases are some of the widely used chemical agents. Detergents lyse cells by permeabilizing and solubilizing cell membranes, while acids and bases solubilize cytoplasmic components and disrupt nucleic acids [43,44]. Biological methods include treating tissues with enzymes, such as proteases and nucleases, and chelating agents. Proteases, such as trypsin, cleave peptides and break cell–matrix adhesions. Nucleases, such as DNase and RNase, catalyze the hydrolytic cleavage of deoxyribonucleotide and ribonucleotide chains [41,45]. Physical methods mainly aim to induce rapid alterations in pressure or temperature to burst the cells, such as high hydrostatic pressure, supercritical carbon dioxide and freeze–thaw cycle [46]. In any case, the decellularization procedure must be gentle to avoid damaging the composition of ECM.

### 3.2. Hydrogel

Hydrogel materials are generally loosely crosslinked polymer materials with a small amount of solid content, and a large amount of water inside. Its 3D structure and biophysical properties (shape, mechanical strength and permeability) are similar to naturally sourced ECM [47]. It is currently extensively used in the medical field with 3D bioprinting, including the repair of skin wounds [48,49,50], bone damage [51,52,53], cartilage injury [54,55,56] and cardiac rehabilitation [57,58,59,60].

Hydrogels are mainly divided into two types: natural polymer hydrogels and synthetic hydrogels. Natural polymer hydrogels, including gelatin, collagen and hyaluronic acid, have the advantages of high biocompatibility, moderate biodegradability, low immunogenicity and long-term stability [60]. Compared with natural polymer hydrogels, synthetic hydrogels have poor biocompatibility, but better mechanical properties [61,62]. 

There is a growing interest on responsive hydrogel with remarkable scientific and technical advances. Responsive hydrogels, one of the hydrogels that can change their physical and chemical properties after being exposed to special external stimuli and respond to them [63,64], have the function of reversibility, self-repair and regeneration, so that they adapt well to the changing external environment. At present, sensitive hydrogels are widely used in the fields of biomedicine and materials, including preparing pH-responsive hydrogels loaded with doxorubicin (DOX) to treat cancer, temperature-responsive hydrogels to make 3D bioprinting materials in wound repair, and glucose-responsive hydrogel in the treatment of diabetic foot [65,66]. In addition, due to the complex internal structures of the human body and the diverse environment of the lesions, single-responsive hydrogels cannot perfectly meet therapeutic expectations under certain pathological conditions [67], such as oxidative stress and pH drop caused by tumors, which involve changes in multiple conditions. Additionally, it is the reason why multiple responsive hydrogels have attracted more and more attention recently, such as temperature-/pH-dual-responsive hydrogels and pH-/redox-dual-responsive hydrogels [68].

### 3.3. D Porous Bioscaffolds

In comparison to dECM and conventional hydrogels, the porous bioscaffolds present more significant advantages for 3D bioprinting. As their surface-interconnected porous structure creates a larger surface area, allowing for the bulk transport of nutrients, waste and biological factors, and also promotes more extensive cell migration and infiltration [69]. Numerous studies are currently attempting to apply 3D porous scaffolds to tissue engineering, for example, in bone regeneration [70,71]. Despite their numerous potential therapeutic applications, the systematic mechanobiological investigation of cellular behaviors in 3D porous bioscaffolds has yet to be performed owing to the lack of methodologies for decoupling the complex interplay between their structural and mechanical properties [72,73]. To settle this problem, Jiang et al. have discovered that the cryoprotectant DMSO can be used to control pore size by regulating ice crystal formation during the fabrication of cryogelated 3D porous scaffolds [72], and the stiffness of the scaffolds was regulated by the degree of crosslinking during cryogelation of the polymer. They chose fibroblasts and macrophages to investigate mechano-responsiveness and achieved separate control of the scaffold pore size and stiffness. Moreover, they explored in vivo cellular responses to these biophysically fine-tuned porous scaffolds after subcutaneous implantation, and our results highlight the potential applications of the scaffolds in improving the therapeutic efficacy of regenerative medicine.

## 4. Three-Dimensional Printing in Each Genitourinary Organ

Mechanics is emerging as a critical hallmark for several diseases and pathologies. Abnormal the mechanical changes and tissue states that occur in disease are important not only for diagnosis, but also for tissue engineering and the recapitulation of such diseases in in vitro models. Regarding living tissues, several mechanical parameters are of clinical significance, and stiffness is considered to be one of the most important and best described mechanical parameters of biological tissues in research. It has become clear that neoplasia, aging and metabolic diseases can lead to fibrosis and an increased rigidity compared to the surrounding healthy parenchyma. Emerging information indicated that the alteration of this mechanical parameter is coupled with the progression of diseases and malignancies associated with kidney fibrosis and cancer, testicular cancer, bladder cancer, prostate cancer and infertility-related diseases [74,75,76]. This information offers new strategies for 3D printing.

Three-dimensional printing technology brings scientific advancement into clinical practice in the field of urology recent years, providing several advantages in surgical planning, resident training, patient education and facilitates the creation of on-demand patient-specific medical devices, implants, or prostheses. Among them, the most common application of 3D printing in the urology field is the creation of patient-specific 3D-printed anatomical and pathological models, as they allow for optimal pre-operative surgical planning, better surgeon understanding of the lesion and ultimately improved patient prognosis [77]. A doctor–patient communication pattern with a 3D-printed organ model helps urologists to obtain patient consent. A survey conducted by Wake et al. concluded that preoperative 3D-printed models help patients to understand their condition and the purpose of the procedure [7]. Three-dimensional-printed models also compensate some of the gaps in traditional cadaver and animal training in urology simulation, providing a low-risk, low-cost opportunity to refine the resident′s professional skills [78]. 

Several studies have also demonstrated the usefulness of 3D-printed models for robotic surgical training [5,79]. Bendre et al. used the Global Assessment of Robotic Skills (GEARS) criteria for assessing resident performance before and after training using a 3D silicone-based kidney model in a robotic pyeloplasty simulation [80]. Additionally, the results found significant improvements in depth perception, surgical speed and confidence after training. In addition, most medical devices are manufactured in standardized combinations of shapes and sizes, which often do not provide the most appropriate product for each patient. Three-dimensional printing allows the creation of medical devices, instruments and tools used in surgery for different patients [81].

## 5. Three-Dimensional Bio-Printing Applications in Urological Tissue Engineering

The urinary system, which consists of the prostate, kidney, ureter, bladder and urethra, is one of the most critical excretory routes for the body′s metabolites, regulating water and salt metabolism and acid–base balance, and producing a variety of biologically active substances that play an important role in maintaining the stability of the body′s internal environment. Patients with structural and functional defects in the tissues of the urinary system require surgical repair and reconstruction or even organ transplantation. However, the reconstructive surgery of the urinary system, unlike destructive surgery, is characterized by greater difficulty, risk and complication rates. Organ transplantation, in turn, is associated with issues such as inadequate supply, immunosuppressive reactions and complications. This has prompted clinicians and scientists to develop new therapies to improve the quality of life of patients with major diseases requiring organ and tissue replacement [82,83]. 

**Figure 3:**
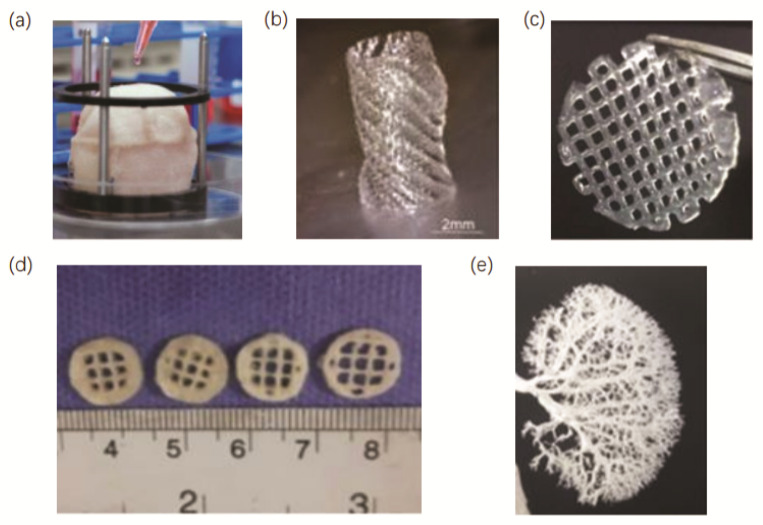
Examples of different urological tissues and organs fabricated by 3D bioprinting: (**a**) 3D-bioprinted bladder model [97]; (**b**) 3D-printed rabbit urethra [91]; (**c**) 3D-bioprinted decellularized vaginal scaffold [92]; (**d**) 3D-bioprinted testicular hydrogel scaffolds [98]; (**e**) 3D-bioprinted rat bionic renal vascular scaffold [96].

Three-dimensional bioprinting allows not only the accurate construction of a precise model of the affected area from the tissue architecture, but also the printing of a variety of different functional cells, extracellular matrix, cell growth factors and biodegradable polymeric support materials. This technology can even reconstruct blood vessels and nerves, achieving the maximum anatomical reset of autologous tissues [84]. In addition, transplantation substitutes can be customized according to the individual differences of patients through 3D printing to achieve the purpose of personalized precision medicine. Currently, this method has been applied to bone repair, cartilage repair, cardiac repair and pelvic repair with promising results; thus, more and more researchers are trying to combine 3D bioprinting with the repair and reconstruction of the urinary system [85,86,87]. In this section, we present some of the recent studies and applications of 3D bioprinting in the repair and reconstruction of the bladder, urethra, testis, vagina and kidney. A summary of recent work on 3D urological bioprinting discussed in this review is presented in Table 3. Additionally, several examples of different urological structures, tissues and organs fabricated by 3D bioprinting are exhibited in Figure 3.

### 5.1. Three-Dimensional Bioprinting of the Bladder

Normal bladder tissue could be constructed into a cellular scaffold by using 3D printing technology, and could eventually create a customized artificial bladder in vitro through cell proliferation [99,100]. The field of urology is currently relatively well researched for the 3D bioprinting of the bladder. Anthony et al. have implanted uroepithelium and muscle cells from seven patients with spinal cord spondylolisthesis onto a biodegradable bladder-shaped scaffold consisting of a composite of collagen and poly-ethanolic acid [88], and then reconstructed the autologous engineered bladder structure and implanted it into the greater omentum wrap of some patients. Urethral and renal function returned without metabolic consequences promptly after surgery. In addition, the engineered bladder biopsies showed adequate structural architecture and phenotype for use in patients requiring cystoplasty. 

**Figure 4:**
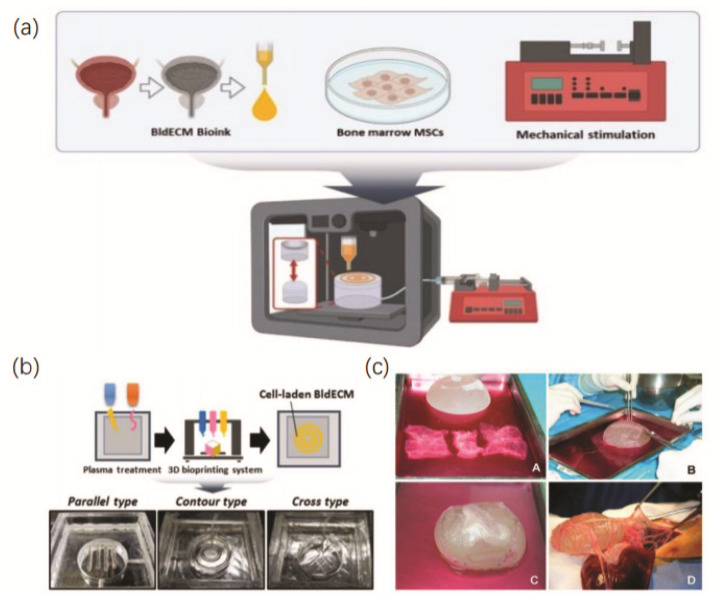
(**a**) Schematic diagram of in vitro 3D-bioprinted bladder model system. Reprinted with permission from ref. [98]; (**b**) Process of 3D-bioprinted bladder of different shapes. Reprinted with permission from ref. [98]; (**c**) Procedure of 3D bioprinting and implantation of bladder bioreactor in vivo. Reprinted with permission from ref. [101].

Chae et al. developed a bladder mimicry platform that incorporates a contract-release system (CRS) using bladder-specific dECM bioink and 3D bioprinting technology that replicate the smooth muscle functions of an actual human urinary bladder [98]. As Figure 4a,b shows, it improved the biological functionality of the 3D bladder tissue in vitro, which could serve as a research platform for fundamental studies on human disease modeling and pharmaceutical testing. Baumert et al. seeded urothelial and smooth muscle cells into a spherical submucosal (SIS) matrix of the small intestine and then transferred them to the great omentum and obtained a bladder in vivo bioreactor [101]. The results showed that, compared with in vitro bioreactor, this in vivo bladder bioreactor loaded with great omentum allows seed scaffolds to mature in vivo with abundant vascularization and has broad application prospects in bladder reconstruction. Its fabrication and implantation process is exhibited in Figure 4c.

Urothelial cells (UC) provide a robust permeability barrier across the urinary tract protects the underlying tissue from toxic components of urine. A compromised urothelium leads to several common urologic diseases, such as urethral injuries or stricture, interstitial cystitis, overactive bladder and bladder cancer. Wang et al. demonstrated that a differentiated urine-derived stem cell (USC)-derived urothelium would provide an excellent platform for the study of interstitial cystitis, overactive, neurogenic or obstructive bladder [102]. 

Nevertheless, there are not sufficient numbers of healthy uroepithelial and smooth cells in all patients available for autologous transplantation. Though mesenchymal stem cells from bone marrow, adipose tissue and skeletal muscle tissue can also be a potential source of cells for autologous transplantation in the urinary system; these cells are more difficult to induce to differentiate into uroepithelial cells. To address this problem, Yang et al. used a rabbit model and discovered that rabbit urine-derived stem cells have a high proliferative and pluripotent differentiation potential and can be easily isolated from urine or bladder washings [103], thus reducing the need for invasive harvesting and cost. They also found that rabbit cells are similar to human urine-derived stem cells in terms of morphology, biochemical properties and differentiation ability, which may also provide a possible direction for the treatment of patients with urethral strictures or urethra defects.

### 5.2. Three-Dimensional Bioprinting of the Urethra

The urethra begins at the internal orifice of the bladder and ends at the external orifice of the urethra, and is responsible for draining urine from the body. Urethral reconstruction tissue engineering is similar to bladder reconstruction cause both of them are achieved using cells extracted from patient biopsies and attached to a synthetic or natural biological scaffold. The urethra of different segments is similar in structure, in which smooth muscle cells and epithelial cells play critical roles. There are few regenerative studies that distinguish between different segments of the urethra.

Atlantida Raya-Rivera et al. have successfully constructed engineered urethras with autologous cells and implanted them into patients with urethral defects [89]. Five boys with urethral defects were included in the study. Firstly, each patient was biopsied through a suprapubic osteotomy to obtain tissue sections, which were cultured to extract muscle and epithelial cells. Then, they implanted these cells onto tubular polyglycolic acid: poly(lactic-co-glycolic acid) scaffolds. After that, patients underwent tissue-engineered urethral tubular reconstruction, and the investigators followed these patients for 72 months. Serial radiographs and endoscopic studies showed that the urethral caliber remained wide without strictures. Urethral biopsy showed that the engineered graft had developed a normal appearance at 3 months post-implantation. Additionally, then they found that the engineered urethra was structurally normal without abnormal histological changes and remained functional in the clinical setting for up to 6 years.

**Figure 5:**
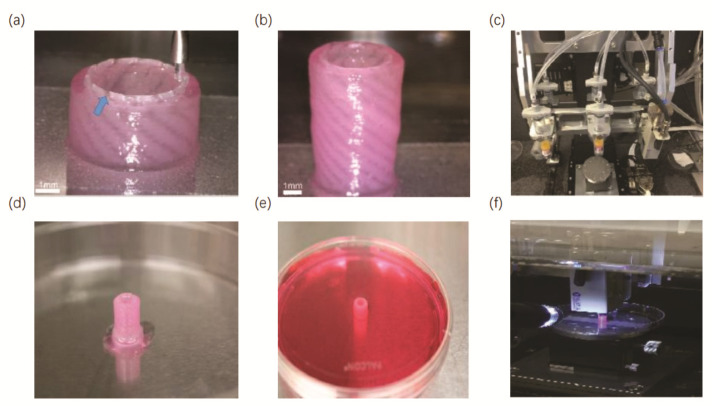
Three-dimensional bio-printing process of rabbit urethra: (**a**) 3D bioprinting using polymers and cell-laden hydrogel; (**b**) 3D-bioprinted rabbit urethra model; (**c**) The 3D bioprinting of scaffold part with polymer nozzle; (**d**) The hydrogel in the urethra model is being cross-linked; (**e**) The urethra model in culture medium; (**f**) The 3D bioprinter with two syringes. Reprinted with permission from ref. [91].

Huang et al. have prepared a novel 3D porous bacterial cellulose (BC) scaffold using a gelatin sponge to interfere with the BC fermentation process and inoculated rabbit lingual keratinocytes derived from lingual mucosa onto the scaffold for implantation into rabbits [90]. The results showed that the scaffold was biocompatible with rabbit lingual keratinocytes and could promote urethral tissue regeneration to a large extent without causing inflammatory reactions, confirming that 3D porous BC with lingual keratinized cells may be a promising alternative scaffold for urethral reconstruction. Zhang et al. have found that the porous spiral scaffold made by PCL and PLCL polymers was highly permeable [91], and their 3D bioprinting process of the urethra is presented in Figure 5. The research has shown that the structural and mechanical properties of the scaffold porous spiral scaffold made by PCL and PLCL polymers were close to those of the natural rabbit urethra, thus facilitating the contact between the printed epithelial cells and the smooth muscle cells on either side of the scaffold. What is more, the bladder epithelium and smooth muscle cells loaded with hydrogel provided a better microenvironment for cellular growth. These results have laid a solid foundation for future research on 3D-bioprinted urethra.

However, most tubular collagen scaffolds used in urological organ reconstruction usually lack the ability to expand radially and reversibly. To address this problem, Versteegden et al. produced a new type of collagen tubular scaffold with inherent radial elasticity [92]. They used the principle of intraluminal folding of tubular hollow organs to introduce the shape recovery effect by in situ fixation using a star-shaped mandrel, 3D-printed clamps and cytocompatible. There is a closed lumen at rest in the prepared stent, and the lumen could open in a star shape when the lumen pressure increases such as when a fluid passes through. In general, the creation of this kind of scaffolds has important implications for the regeneration of tubular organs in the urinary system.

### 5.3. Three-Dimensional Bioprinting of the Testis

There is relatively fewer research on 3D bioprinting technology for the testis. For patients with testicular agenesis, small bilateral testes and severe bilateral testicular atrophy, their treatment is based on autologous or allogeneic transplantation. Some studies suggested that chemotherapy and radiotherapy used for childhood cancer treatment can irreversibly affect fertility in adulthood, and cryopreserved immature testicular tissue transplantation (ITT) may be a promising strategy to restore fertility in young boys for this group of patients since prepubertal males do not produce sperm. However, the number of spermatogonial cells was significantly reduced after ITT transplantation regardless of the cryopreservation approach [72]. To address this issue, Poels et al. embedded ITT in hydrogels loaded with VEGF nanoparticles and assessed seminiferous tubule integrity, haematopoietic reconstitution and spermatogonia recovery by immunohistochemistry [93]. The results revealed that the alginate hydrogels containing nanoparticle growth factors had a significantly higher spermatogonia recovery and had the potential to facilitate cryopreservation in terms of tissue transplantation.

Some studies suggest that testicular stents may be an alternative method to construct artificial testis and preserve fertility in patients. Zahra et al. have printed a testicular hydrogel scaffold by decellularizing the testicular tissue fragments of RAMS and combining the extracted T-ECM with alginate and gelatin [99]. Then, they characterized the morphology, mechanical properties and biological properties of the scaffolds. The results showed that the hydrogel containing 5% ECM was the most suitable scaffold for testicular cell culture, with strong cell adhesion and high cell biocompatibility for spermatogonial stem cells. Such scaffolds can be used as a biomimetic material for the preparation of artificial testis, which may have certain application significance in reproductive medicine. The post-transplant collagen scaffolds provide high biocompatibility without any signs of inflammation that fully compliant with clinical use requirements. It is of great importance to perform more studies to confirm ways of further limiting the loss of spermatogonia, while demonstrating their ability to differentiate.

### 5.4. Three-Dimensional Bioprinting of the Vagina

It is an urgent need for vaginal reconstruction for patients whose congenital absence, vaginal atresia, and vaginal stenosis or injury have been suffering great psychological and physical pain. Traditional vaginal reconstruction techniques use non-vaginal tissue, which is morphologically and histologically very different from the normal vagina and thus suffers from low cell viability and crude construction. Fortunately, 3D bioprinting holds the promise of solving these problems. Hou et al. transformed the porcine acellular vaginal matrix (AVM) with a mixture of 15% gelatin and 3% sodium alginate into bioink and characterized its viscosity and morphology [94]. Additionally, researchers then embedded bone marrow mesenchymal stem cells in the 3D scaffold and transplanted them into rats. The results showed that the bionic 3D vaginal tissue exhibited good epithelialization, vascularization and biocompatibility.

### 5.5. Three-Dimensional Bioprinting of the Kidney

The kidneys are paired lentil-shaped organs, reddish-brown in color, located in shallow fossae on either side of the retroperitoneal spine. This organ is made up of more than one million kidney units, each consisting of three parts: the glomerulus, the renal capsule and the renal tubules. The basic function of the kidneys is to produce urine to remove metabolites and certain waste products and toxins from the body, and to retain water and other useful substances through reabsorption to regulate water and electrolyte balance and maintain acid–base balance. The kidneys also have an endocrine function, producing renin, erythropoietin, active vitamin D3, prostaglandins, etc. However, the incidence of acute or chronic kidney disease is increasing recently due to dietary problems, urinary tract infections and the increased use of prescription drugs, which continues to prompt researchers to explore the construction of three-dimensional structures of the kidney to improve, restore or replace some or all of its functions [96,104,105].

The convoluted proximal tubule is one of the most vulnerable compositions in the kidney, as the proximal tubule is involved in the re-absorption of glomerular filtrate and the secretion of metabolites, leading to high concentrations of drug metabolites accumulating in the proximal tubule. Kimberly et al. have successfully constructed proximal tubules in the kidney in vitro using 3D printing technology [95]. These convoluted proximal tubules consist of an open lumen architecture circumscribed by proximal tubule epithelial cells (PTECs), embedded in an extracellular matrix made by gelatin–fibrin hydrogels, and housed within a perfusable tissue chip, where they are subjected to physiological shear stresses. Scanning electron microscopy and a series of biochemical reactions have shown that these artificial proximal tubules have essentially the same physiological function as normal human proximal tubules. To sum up, these 3D-printed tubules can be used to analyze drug metabolism and assist in vitro dialysis.

Little et al. have created renal micro-organs, which provide an economical source of renal cell production [105]. However, continuous improvements in size, structure and function are required to achieve the goal of alternative renal tissue. Among them, vascularization is one of the major hurdles affecting the survival and integration of implanted 3D kidney constructs in vivo. The kidney depends on complex three-dimensional vascular networks to maintain its normal physiological activity, but the intricate nature of these networks’ net makes the replication of native vessels difficult.

To address this challenge, Jennifer et al. have developed a bionic renal vascular scaffold based on the vascular corrosion casting technique [98]. They perfused rat kidneys with 10% polycaprolactone (PCL) and then followed by tissue digestion. The cast surface of the corroded PCL was covered with collagen and the PCL was then removed from within the collagen coating, leaving only a hollow collagen-based bionic vascular scaffold. After that, the prepared scaffolds were pre-vascularized with an MS1 endothelial cell coating and then bonded to the 3D kidney structure and implanted into the renal cortex of nude rats. The results showed that this novel vascular scaffold effectively improved vascularization, and the addition of human kidney cells to the MS1-coated scaffold further enhanced the vascularization and regeneration of renal tubular structures within the implanted scaffold. Accordingly, the use of this scaffold could greatly address the challenges associated with vascularization and maybe an ideal therapeutic strategy treatment for the partial augmentation of renal function in patients with chronic kidney disease.

Although 3D bioprinting could theoretically produce artificial kidneys for transplantation, researches in this area are still in its infancy due to the more complex composition and structure of the kidney compared to the bladder and urethra.

## 6. Challenges and Perspectives

Despite the achievements of 3D bioprinting technology over the past decades, there are still many challenges in the manufacturing of organs and tissues in urological applications. Firstly, 3D data reconstruction must be accurate and 3D bioprinting costs are generally high, which makes it difficult to achieve widespread application. Secondly, it is relatively difficult to cultivate and acquire seed cells for printing, and technicians with rich experience in cell culture are needed. Thirdly, the support materials used for printing should not only have good biocompatibility and biomechanical properties, but also meet the specific structural requirements of the urinary system. Fourthly, special 3D printers and nozzles are needed to keep cells and cytokines alive. Finally, the preparation of functional 3D-bioprinted tissue containing blood vessels is still one of the challenges faced by 3D bioprinting. Therefore, further research and exploration are needed to exploit the advantages of 3D bioprinting technology and to make new transplant substitutes available for clinical use [1,2,17].

## 7. Concluding Remarks

Three-dimensional bioprinting technology is based on computer 3D models and uses bioprinting materials, such as dECM, hydrogels and growth factors, to print the required structurally complex and fully functional biomedical products by discrete stacking following the bionic morphology function and environment. It has potential development prospects and a wide range of clinical applications through the combination of manufacturing technology and life sciences. At the same time, 3D bioprinting technology is also a multidisciplinary cross-application technology, requiring the integration of cell biology, computers, materials, information, chemistry, mechanics, engineering, manufacturing, medicine and other scientific and technological fields of talent. At present, there are still some technical difficulties in 3D bioprinting technology, and only through continuous research breakthroughs will it be possible to apply 3D bioprinting to a wide range of clinical applications and bring benefits to patients [6,15,16,106,107].

## Figures and Tables

**Table 1:** Comparison of currently common 3D bioprinting technologies.

Bioprinting Methods	Cell Viability	Ink Viscosity	Printing Speed	Related Costs	Resolution
**Micro-extrusion**	40–95%	Wide range	Low	Moderate	Low
**Inkjet**	>85%	Very low	High	Low	Moderate
**Laser-assisted**	>95%	Low	Moderate	High	High

**Table 2:** Comparison of currently common 3D bioprinting bioinks.

Type	Composition	Classification	Characteristics
**dECM**	The fraction obtained by removing the cellular components and some small molecules or antigens from the natural ECM	Animal-derived; Human-derived; Plant-derived	Excellent biocompatibility; Good degradability and immunogenicity; nutrient-rich
**Hydrogel**	Extremely hydrophilic three-dimensional network structure gel	Natural polymer hydrogels; Synthetic hydrogels	High biocompatibility; Low immunogenicity; Long-term stability; Responsive hydrogels
**3D porous** **bioscaffold**	A novel scaffold with micron or even nanopore structure	Natural sources; Synthetic	Large surface area; Greatly facilitates material transport and cell attachment

**Table 3:** Urological 3D Bioprinting Projects, Their Printing Techniques, and Bioink Preparation.

Field	Research Goal	3D Bioprinting Technique	Scaffold Biomaterial	Cell Type	Reference
**Bladder**	Development of an alternative approach using autologous engineered bladder tissues for reconstruction	Multicellular spheroid formation	Collagen; Polyglycolic acid	Human uroepithelial and muscle cells	[88]
**Urethra**	Assessment of the effectiveness of tissue-engineered urethras using patients’ own cells in patients who needed urethral reconstruction	Multicellular spheroid formation	Lactide-co-glycolide acid	Human smooth muscle and urothelial cells	[89]
**Urethra**	Evaluation of the effects of urethral reconstruction with a three-dimensional (3D) porous bacterial cellulose (BC) scaffold seeded with lingual keratinocytes in a rabbit model	Multicellular spheroid formation	3D porous bacterial cellulose	Rabbit lingual keratinocytes	[90]
**Urethra**	Construction of 3D bioprinting urethral using PCL, PLCL and different rabbit cell types	Inkjet	PCL; PLCL	Rabbit urothelial cells and smooth muscle cells	[91]
**Urethra**	Construction of a new type I collagen-based tubular scaffold is presented that possesses intrinsic radial elasticity	Extrusion-based	Insoluble type I collagen; Carbodiimide crosslinking	SCaBER cells	[92]
**Testis**	Development of the potential of alginate hydrogel loaded with nanoencapsulated growth factors to Improve cryopreserved tissue engraftment	Tissue encapsulation	VEGF nanoparticles Alginate; Fibrin	Spermatogoni-al	[93]
**Vagina**	Reconstruction of the biomimetic 3D vagina tissue with AVM bioink encapsulating BMSCs	Inkjet	Acellular vagina matrix; Sodium; Gelatin	Bone marrow mesenchymal stem cells	[94]
**Kidney**	Construction of a bioprinting method for creating 3D human renal proximal tubules in vitro	Inkjet	Fibrinogen; Gelatin	PTEC-TERT1 cells	[95]
**Kidney**	Kidney regeneration with biomimetic vascular scaffolds based on vascular corrosion casts	Embedding and coating	Hollow collagen vascular scaffold	MS1 cells, Human renal cells	[96]
